# Impaired in vitro growth response of plasma-treated cardiomyocytes predicts poor outcome in patients with transthyretin amyloidosis

**DOI:** 10.1007/s00392-020-01801-y

**Published:** 2021-01-22

**Authors:** Selina Hein, Jennifer Furkel, Maximilian Knoll, Fabian aus dem Siepen, Stefan Schönland, Ute Hegenbart, Hugo A. Katus, Arnt V. Kristen, Mathias H. Konstandin

**Affiliations:** 1 Department of Cardiology, Angiology and Pulmonology, University Hospital Heidelberg, INF 410, 69120 Heidelberg, Germany; 2 DZHK (German Center for Cardiovascular Research), Site Heidelberg/Mannheim, Heidelberg, Germany; 3 Department of Radiation Oncology, Heidelberg Ion-Beam Therapy Center (HIT), German Cancer Research Center, Heidelberg University Hospital (UKHD), Heidelberg, Germany; 4 Department of Hematology, Oncology and Rheumatology, Heidelberg University, Heidelberg, Germany

**Keywords:** Transthyretin, Amyloidosis, In-vitro assay, Hypertrophy

## Abstract

**Objectives:**

Direct toxic effects of transthyretin amyloid in patient plasma upon cardiomyocytes are discussed. However, no data regarding the relevance of this putative effect for clinical outcome are available. In this monocentric prospective study, we analyzed cellular hypertrophy after phenylephrine stimulation in vitro in the presence of patient plasma and correlated the cellular growth response with phenotype and prognosis.

**Methods and results:**

Progress in automated microscopy and image analysis allows high-throughput analysis of cell morphology. Using the InCell microscopy system, changes in cardiomyocyte’s size after treatment with patient plasma from 89 patients suffering from transthyretin amyloidosis and 16 controls were quantified. For this purpose, we propose a novel metric that we named Hypertrophic Index, defined as difference in cell size after phenylephrine stimulation normalized to the unstimulated cell size. Its prognostic value was assessed for multiple endpoints (HTX: death/heart transplantation; DMP: cardiac decompensation; MACE: combined) using Cox proportional hazard models. Cells treated with plasma from healthy controls and hereditary transthyretin amyloidosis with polyneuropathy showed an increase in Hypertrophic Index after phenylephrine stimulation, whereas stimulation after treatment with hereditary cardiac amyloidosis or wild-type transthyretin patient plasma showed a significantly attenuated response. Hypertrophic Index was associated in univariate analyses with HTX (hazard ratio (HR) high vs low: 0.12 [0.02–0.58], *p* = 0.004), DMP: (HR 0.26 [0.11–0.62], *p* = 0.003) and MACE (HR 0.24 [0.11–0.55], *p* < 0.001). Its prognostic value was independent of established risk factors, cardiac TroponinT or N-terminal prohormone brain natriuretic peptide (NTproBNP).

**Conclusions:**

Attenuated cardiomyocyte growth response after stimulation with patient plasma in vitro is an independent risk factor for adverse cardiac events in ATTR patients

**Supplementary Information:**

The online version contains supplementary material available at 10.1007/s00392-020-01801-y.

## Introduction

Amyloidosis comprises a group of rare diseases that are caused by deposition of amyloid fibrils in tissue and consecutively induce progressive organ dysfunction. One of the most frequent types of amyloidosis is transthyretin amyloidosis (ATTR). Hereditary ATTR results from various point mutations in the transthyretin gene (ATTRv) leading to reduced stability of the TTR tetramer due to protein misfolding, which ultimately will be deposed in the respective organs. Primarily involved organs are the heart and peripheral nerves. Moreover, non-hereditary, wildtype TTR amyloidosis (ATTRwt) exists in elderly mainly male patients without any germline mutation and primarily affects the heart [1, 2].

In the very recent past, direct toxic effects of amyloid upon cardiomyocytes have been proposed mainly for light chain amyloidosis (AL) but also for ATTR [3–5]. In general the heart as post-mitotic organ reacts to increased work load demand and stress with hypertrophic growth. However, this adaptive response soon contributes to the progression of the underlying disease [6]. In clinical routine natriuretic peptides and cardiac troponins are surrogates of increased cardiac stress correlating well with the prognosis of the disease [7, 8].

Miniaturization to 96-well plates as well as progress in automated image acquisition and analysis in microscopy has opened the possibility to quantify cell size for in vitro experimentation in high-throughput approaches or translational studies [9, 10]. With all limitations regarding species and effort to prepare primary cells, the best cell model to study cardiac hypertrophic response in vitro are neonatal rat cardiomyocytes (NRCM). The well-established positive control for hypertrophic response in vitro represents phenylephrine (PE) treatment [11, 12]. Therefore, in the herein study we analyzed the hypertrophic growth of NRCMs upon stimulation with two concentrations of plasma from patients with ATTR amyloidosis. Furthermore, the reactivity towards additional PE treatment was quantified. Results were correlated with the clinical phenotype (ATTRv-PN, ATTRv-CA, ATTRwt) and tested for their prognostic value during a median follow-up period of 14.8 months.

## Methods

### Study population

Between October 2016 and November 2017, patients who consecutively presented in our tertial referral center for amyloidosis at Heidelberg University Hospital were screened to participate in this study. 91 fulfilled the inclusion criteria and subscribed written informed consent approved by the ethical review committee Heidelberg and in accordance with the declaration of Helsinki. Inclusion criteria comprised age > 18 and < 90 years, diagnosis of ATTRv, ATTRwt or asymptomatic carrier of an ATTRv. According to genotype and the clinically leading organ manifestation, ATTRv patients were subdivided into patients with predominantly cardiac phenotype (ATTRv-CA) and patients with predominantly neuropathic phenotype (ATTRv-PN). This latter group consisted of *n* = 23 patients with the Val30Met mutation, which was initially described as the prototype of amyloidosis with neurological manifestation [13–15]. In our cohort only six patients did not show cardiac manifestation. Exclusion criteria were liver transplantation, participation in interventional clinical trial or TTR expression modifying therapy (e.g. inotersen or patisiran therapy). Patients who were under TTR stabilizer therapy, however, were admitted to participate at the study (*n* = 24). Blood samples were attained in the course of a routine venopuncture. One additional lithium heparin monovette (4.9 ml, Sarstedt) was collected and used for his study. Healthy volunteers were recruited to build up a control group. To be eligible for the control group, clinical presentation, echocardiography and biomarkers (C-reactive protein (CRP), cardiac troponin T (cTnT) and N-terminal prohormone brain natriuretic peptide (NTproBNP)) needed to be within the normal range. After the inclusion of the last patient, in vitro cell size analysis was performed in three independent experiments. For subgroup analysis in Fig. 3, patients were divided into patients with positive cTNT levels according to the estimated cutoff in our recent study [16]. For subcohort definition regarding natriuretic peptide levels (glomerular filtration rate (GFR) adjusted NTproBNP), measured NTproBNP levels were adjusted to renal function as described by Luchner et al. (NT-proBNP_adjusted_ = NT-proBNP_measured_/e^(1.892–0.025×GFR)^) [17]. The cutoff levels for NTproBNP positivity were chosen adjusted for age according Hildebrandt et al. [18].

### Follow-up

Endpoint follow-up was performed by interviewing patients directly during outpatient visits or via phone call after 24 months. Additionally, patient files of subsequent hospitalization were analyzed. Median follow-up time was 14.8 [95% CI 14.2–16.9] months. Endpoints were (1) death of any cause or cardiac transplantation (HTX), (2) hospitalization due to cardiac decompensation (DMP) and (3) combined endpoint consisting of any of the major cardiovascular events from (1) or (2) (MACE).

### Cell culture

We isolated neonatal rat cardiomyocytes (NRCMs) as described elsewhere [19]. Briefly, after trypsin isolation of NRCMs we transferred the cells to 96-well cell culture plates (Falcon, Clear Flat Bottom TC-Treated Imaging Plate with Lid 353,219, corning), which were pre-coated with gelatin for 2 hours. After removal of coating medium, 20,000 cells per well were transferred to the cell culture plate. During the first 24 h cells were cultured using Dulbecco’s modified eagle Medium (DMEM/F12) including 10% fetal calf serum (FCS). The next day, when NRCMs had adequately attached, cell culture plates were rinsed three times with 37 °C warm phosphate-buffered saline (PBS) using a multichannel pipette (Eppendorf 100 µl). Afterwards cells were treated with 5% or 20% plasma in DMEM/F12 medium, without and with additional phenylephrine (PE) stimulation (50 µM). These cell culture conditions were chosen from our experience with NRCM experimentation and are in the range of doses applied by other groups for in vitro experiments [20, 21]. Each condition was evaluated in quadruplets in three independent experiments (NRCM preparations on different days). Fetal calf serum-treated cells were included on each analyzed plate as control for normalization per plate. After 48 h at standard conditions in the incubator (37 °C, 5% CO_2_) treatment was stopped by washing the cells with PBS and fixation using 4% paraformaldehyde (PFA) for 30 min.

### Immunohistological staining

Fixed cells were washed three times with PBS and permeabilized using 0.1% triton solution for 5 min [19]. Cells were washed one time and plates were incubated with RNase (RNase A 100 mg/ml, 7000 U/ml, Qiagen) in 1:1000 dilution using PBS for 15 min at room temperature. Then plates were washed with PBS and exposed to blocking solution (PBS + 10% FCS) for 1 h at room temperature. Plates were incubated with a polyclonal Desmin anti rabbit antibody (ab15200, Abcam) overnight at 4 °C to stain cellular body. Primary antibody was detected by the goat anti rabbit antibody Alexa Fluor^®^ 594 preadsorbed (ab150088, abcam). Nuclear DNA was stained using 4′,6-diamidino-2-phenylindole (DAPI).

### Cell size analysis

Cell size was quantified automatically, after image acquisition with the In Cell Analyzer 2200 (GE Healthcare Life Science). 16 images were taken per well. Image analysis was performed using Cell Profiler Version 2.2.1 [22]. Single cell measurements underwent image-based quality checks for vitality of cells based on adequate DAPI intensity and ratios of cellular to nuclear size prior to aggregation per well (≥ 1.5). Three independent experiments were conducted to obtain replicates (4 wells per replicate). After filtering steps, for two patient samples, only two replicates were retained, in six samples, only results from one experiment was used for further analysis. For the complete process of automated data acquisition and analysis of cell size authors were fully blinded.

### Statistical analysis

**Fig. 1:**
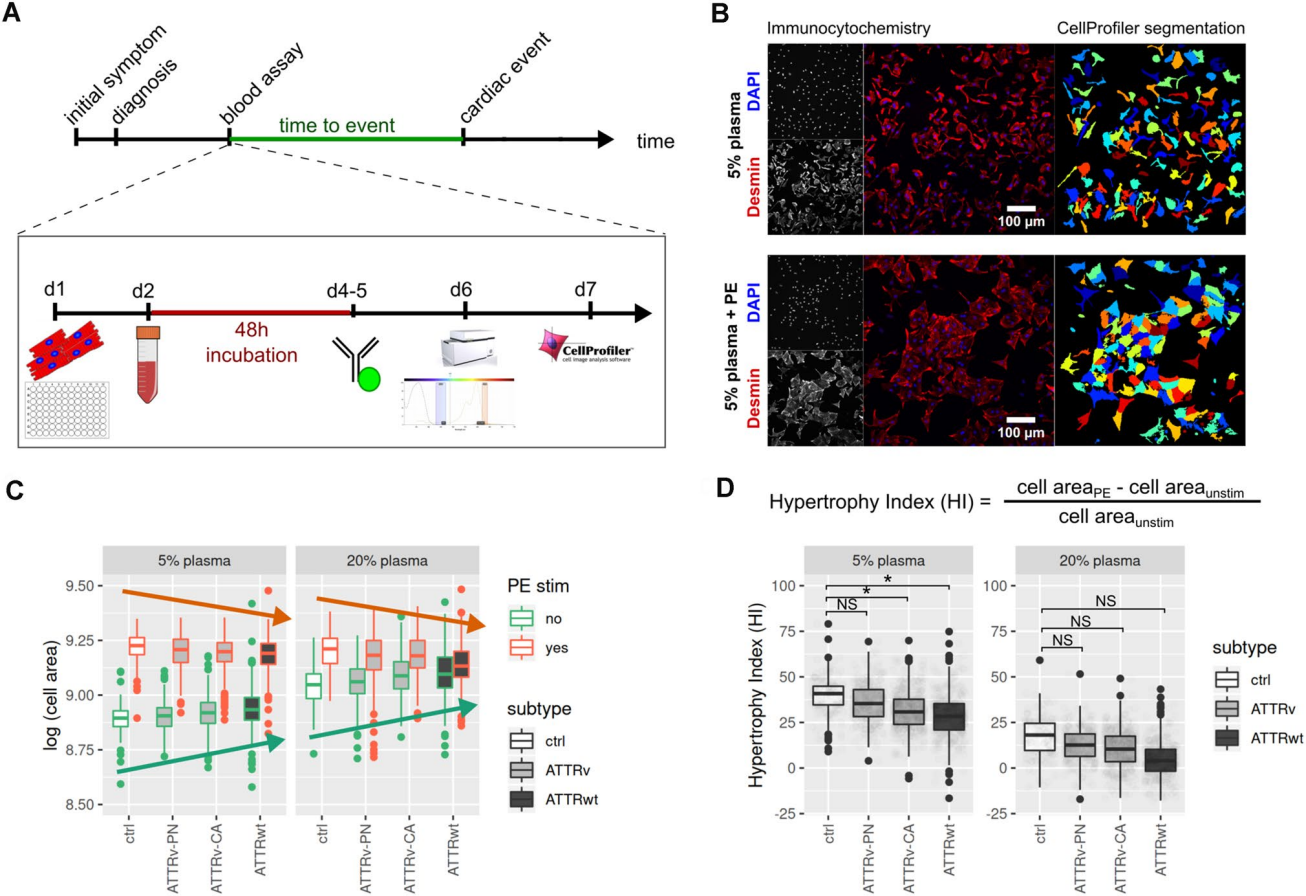
Overview of the experimental setup. **a** Timeline and Assay workflow is depicted. Blood plasma from patients was collected during routine visit, and events during follow-up (decompensation, transplantation and dead) were prospectively documented. After seeding cardiomyocytes at day 1, the culture medium was replaced after 24 h (h) with 5% or 20% patient plasma and treated additionally with or without phenylephrin. After another 48 h the cellular growth was stopped by fixation and the cells were stained as described in the “Methods” section. Data acquisition and analysis was performed in an automated setup. **b** Representative immunohistological specimen (left) are shown for NRCMs incubated with 5% human control plasma without (top) or with additional phenylephrine (PE) treatment (bottom). Cells were stained with Desmin antibody (red) and DAPI for nuclei detection (blue). The corresponding automated area recognition by the software is shown on the right. **c** Quantification of cell size after plasma treatment (5% left; 20% right) for unstimulated condition (green frame of the box plots) and after PE treatment (red frames of the box plots) is shown for controls and ATTR amyloidosis patients (ATTRv-PN, ATTRv-CA, ATTRwt). The red arrow illustrates a slight decrease of cell size after PE stimulation in between the groups. The green arrow illustrates a slight increase of cell size under basal condition in between the groups **d** The formula for hypertrophy index (HI) calculation is depicted on top. HI for 5% and 20% plasma stimulation is shown below. “Star” indicates significantly different values, *p* < 0.05, NS: indicates not significantly different values. Please note: for illustrative reasons data are presented as relative cell size. For statistics, however, log-transformed values were used. Data were attained from three independent experiments

Statistical analyses were conducted using R, v3.5.1 [23]. Cell sizes were normalized per plate to FCS controls. Quantification of cell size changes was performed by evaluating the hypertrophic index (HI), the ratio of differences (PE stimulated vs. non-stimulated) to non-stimulated cell sizes after log transformation. Also for Cox regression analysis this log transformed index was used. For demonstration purposes, ratios were also calculated for non-log transformed data, as shown in Fig. 1d as relative increase in cell size. Uni- and multivariate survival analyses were conducted with Cox proportional hazard models (survival package [24]); *p* values derived from likelihood ratio tests are reported for Kaplan–Meier curves and from Wald tests for uni/multivariate analyses. Cutoff selection for prognostic stratification of patients based on the HI variable (aggregated per well, up to 12 measurement per sample) was conducted using the *dataAnalysisMisc* package [25] while adjusting for correlation between samples. The combined endpoint was used for threshold detection. Patients were classified into “high”/“low” HI groups based on median values of well-wise measurements as compared to the determined cutoff. The optimal cutoff was calculated by testing incremental cutoffs to minimize the observed *p* value for prognostic separation. Differences in cell sizes were evaluated using mixed effect models (*nlme* [26]) on cell sizes (per well), using patient IDs as random factor. Associations between diagnosis groups and patient characteristics were evaluated using ANOVA or chi-squared tests for categorical and continuous variables, respectively. Median follow-up differences were calculated using a Cox-PH model. Test-results with *p* values below *α* = 0.05 were considered significantly different.

## Results

### Study population

A total of 89 patients and 16 controls were included in the present study. After genotyping, patients were grouped into ATTRwt (*n* = 30), ATTRv-CA (*n* = 36) und ATTRv-PN (*n* = 23) according to the clinically leading symptoms [27]. The ATTRv-CA group consists of 36 patients [Val20Ile (*n* = 13) Cys10Arg (*n* = 5), Val122Ile (*n* = 4), Leu58His (*n* = 5), Ile107Val (*n* = 5), Thr126Arg (*n* = 1), Ile84Asn (*n* = 1), Ile84Thr (*n* = 1), Ala45Thr (*n* = 1)]. Among those, 11 patients were asymptomatic mutation carrier [Val20Ile (*n* = 7), Val122Ile (*n* = 1), Cys10Arg (*n* = 1), Ile107Val (*n* = 1), Ile84Thr (*n* = 1)]. Patients who presented with a mixed phenotype or solely polyneuropathy were classified into the group ATTRv-PN. In this group we included 23 patients with Val30Met mutation. Out of those, six were cardiac asymptomatic mutation carriers. Table 1 shows clinical characteristics of all patients. ATTRwt patients were older and significantly more common male patients. Furthermore, treatment with ACE inhibitors, AT1 antagonists, calcium channel blockers, diuretics as well as beta blockers was more common in ATTRwt patients. More ATTRwt patients were equipped with a pacemaker and basic rhythm was more often atrial fibrillation compared to both other patient groups. Bundle branch blocks were more common, cTnT levels were significantly higher and glomerular filtration rate (GFR) was reduced in ATTRwt patients compared to the other groups. All other variables tested (BMI, medication, Karnofsky index, diabetes, times in the ECG, echocardiographic parameters, NTproBNP levels) were not significantly different between groups.

**Table 1:** Patient characteristics: differences in patient groups were assessed using analysis of variance for continuous variables (ANOVA) and Chi Square Tests for categorical data

	ATTRv-CA (*n* = 36)	ATTRv-PN (*n* = 23)	ATTRwt (*n* = 30)	Control (*n* = 16)	*p* value
Age (years)	59.4 ± 1.6	57.5 ± 3.2	76.3 ± 1.2	50.6 ± 3.7	< 0.001
Sex					0.022
Male	26 (72.2%)	17 (73.9%)	26 (86.7%)	7 (43.8%)	
Female	10 (27.8%)	6 (24.0%)	4 (13.3%)	9 (56.3%)	
BMI	26.8 ± 5.7	25.0 ± 4.1	25.5 ± 2.9	26.7 ± 6.7	0.3
**Medication**					
Tafamidis	11 (30.6%)	13 (56.5%)	0 (0.0%)	0 (0%)	0.68^§^
Beta blocker	10 (27.8%)	7 (30.4%)	25 (83.3%)	3 (18.8%)	< 0.001
ACE inhibitors/AT1 antagonists	13 (36.1%)	4 (17.4%)	21 (70.0%)	3 (18.8%)	< 0.001
Diuretics	16 (44.4%)	7 (30.4%)	29 (96.7%)	2 (12.5%)	< 0.001
Other antihypertensive medication (amlodipine, doxazosin, nitrendipine)	2 (5.6%)	2 (8.7%)	6 (20.0%)	0 (0.0%)	0.06
**Functional impairment**					
Karnofsky performance index					0.18
≥ 80	33 (91.7%)	16 (69.6%)	17 (56.7%)	16 (100%)	
< 80	3 (8.3%)	4 (17.4%)	1 (3.3%)	0 (0%)	
NYHA class					< 0.001
I	17 (47.2%)	11 (47.8%)	1 (3.3%)	14 (87.5%)	
II	11 (30.6%)	6 (26.1%)	13 (43.3%)	2 (12.5%)	
III	5 (13.8%)	0 (0%)	15 (50%)	0 (0)	
IV	1 (2.8%)	0 (0%)	1 (3.3%)	0 (0)	
**Medical history**					
Pacemaker implantation	4 (11.1%)	3 (13.0%)	6 (20.0%)	0 (0.0%)	0.12
Carpal tunnel syndrome	18 (50.0%)	12 (52.2%)	16 (53.3%)	0 (0.0%)	0.54^§^
Diabetes	3 (8.3%)	0 (0.0%)	6 (20.0%)	0 (0.0%)	0.01
Atrial fibrillation	8 (22.2%)	5 (21.7%)	22 (73.3%)	2 (12.5%)	< 0.001
**ECG findings**					
Number of bundle branch	0.7 ± 0.8	0.7 ± 0.9	1.2 ± 0.7	0.3 ± 0.6	0.03
Sinus rhythm	30 (83.3%)	17 (73.9%)	14 (46.7%)	15 (93.8%)	0.03
Atrial fibrillation	5 (13.9%)	4 (17.4%)	13 (43.3%)	1 (6.3%)	0.003
Pace maker rhythm	1 (2.8%)	1 (4.3%)	3 (10.0%)	0 (0.0%)	0.28
Low voltage pattern	6 (16.7%)	3 (13.0%)	8 (26.7%)	0 (0.0%)	0.03
Heart frequency (bpm)	71.3 ± 13.7	70.5 ± 25.0	77.4 ± 13.4	68.4 ± 9.2	0.94
PQ (ms)	167.4 ± 38.3	166.2 ± 36.6	215.1 ± 35.1	159.7 ± 27.1	0.61
QRS (ms)	107.0 ± 25.4	111.1 ± 30.1	128.2 ± 36.1	96.5 ± 11.0	0.69
QTc (ms)	423.4 ± 34.7	388.3 ± 117.6	446.7 ± 34.1	402.6 ± 9.3	0.15
**Echocardiography**					
IVS (mm)	15.5 ± 4.9	13.9 ± 6.4	19.2 ± 4.7	11.4 ± 1.3	0.43
HW (mm)	13.3 ± 4.0	11.1 ± 4.2	15.7 ± 3.4	9.6 ± 1.0	0.45
Ejection fraction(%)	51.3 ± 10.0	53.0 ± 9.8	41.2 ± 10.5	56.4 ± 8.7	0.48
Diastolic dysfunction	23 (63.9%)	14 (60.9%)	27 (90.0%)	2 (12.5%)	
Strain	13.5 ± 6.4	14.2 ± 3.9	7.6 ± 5.8	21.0 ± 3.5	0.74
MAPSE (cm)	1.2 ± 0.5	1.8 ± 2.8	0.8 ± 0.2	1.3 ± 0.4	0.81
TAPSE (cm)	1.9 ± 0.6	2.4 ± 2.5	1.3 ± 0.5	2.0 ± 0.5	0.41
Pericardial effusion	3 (8.3%)	1 (4.3%)	4 (13.3%)	0 (0%)	0.17
PA pressure (mmHg)	30.7 ± 8.4	34.1 ± 11.3	41.0 ± 10.2	27.5 ± 3.5	0.57
**Biomarkers**					
NTproBNP (pg/ml)	1451.3 ± 328.9	1427.3 ± 457.4	4254.1 ± 561.9	117.3 ± 75.6	0.11
hsTnT (ng/l)	51.9 ± 19.8	20.9 ± 11.0	67.1 ± 6.8	7.2 ± 2.7	< 0.001
GFR	78.5 ± 26.7	87.0 ± 25.4	52.6 ± 15.9	91.6 ± 24.3	< 0.001
**Follow up**					0.01
Median FU (95% CI)	14.8 (13.90–16.9)	14.8 (13.67–16.9)	21.2 (17.12–25.9)	10.5 (4.79–14.2)	

Mean ± SED values are reported if not 457 indicated otherwise

^§^Only ATTRv-CA vs ATTRv-PN

^#^Time point of blood drawing

### Hypertrophic growth due to phenylephrine treatment is attenuated in ATTRv-CA- and ATTRwt-plasma treated NRCMs

The study protocol is shown in Fig. 1a. After completion of patient recruitment, NRCMs were treated with patient plasma with and without additional PE stimulation. Representative images (Fig. 1b) of NRCMs treated with 5% plasma from control subjects are shown without (upper row) or with additional PE treatment (lower row). Automated cell area recognition by the software is depicted next to the immunohistological specimen in Fig. 1b on the right. Cell sizes were then automatically quantified. Quantified cell sizes for all subjects are shown in Fig. 1c after treatment with 5% plasma (left) and 20% plasma (right). Although not significantly different, in trend cell sizes increased from control over ATTRv-PN and ATTRv-CA to ATTRwt in the unstimulated condition in the presence of 5% as well as 20% plasma (green arrow, Fig. 1c). In contrast, upon PE stimulation cellular growth response was in trend impaired in cells treated with patient plasma compared to healthy controls (red arrow, Fig. 1c). Under the 5% plasma condition within each group, PE treatment led to a considerable increase of cell size compared to the respective unstimulated condition; this effect was less pronounced under the 20% plasma condition (Table 2).

**Table 2:** Univariate time to event analyses: Cox proportional hazard models, Wald* p* values are reported

	Feature	HR (95% CI)	*p* value	*n*/events
Combined endpoint	Cell size (dichotom), high vs low	0.24 (0.11–0.55)	< 0.001	105/25
	NTproBNP, log	1.15 (0.92–1.45)	0.23	89/20
	NTproBNP, GFR adjust, log	2.02 (1.40–2.91)	< 0.001	95/25
	TnT, log	3.01 (1.89–4.78)	< 0.001	98/25
	GFR	0.96 (0.94–0.97)	< 0.001	102/25
	Age	1.06 (1.02–1.10)	< 0.001	105/25
	Sex, male vs female	4.32 (1.02–18.35)	0.047	105/25
	Group, ATTRv-PN vs ATTRwt	0.62 (0.20–1.93)	0.41	105/25
	Group, ATTRv-CM vs ATTRwt	0.87 (0.38–2.02)	0.75	
	MAPSE, log	0.38 (0.13–1.13)	0.08	93/25
	EDD	0.99 (0.92–1.07)	0.83	94/25
	Posterior wall	1.24 (1.12–1.37)	< 0.001	93/25
	Intraventricular septum	1.14 (1.06–1.22)	< 0.001	94/25
	Left ventricular hypertrophy index	12.39 (3.18–48.3)	< 0.001	93/25
Decompensation only	Cell size (dichotom), high vs low	0.26 (0.11–0.62)	0.003	105/21
	NTproBNP, log	1.17 (0.90–1.52)	0.24	89/16
	NTproBNP, GFR adjust, log	2.17 (1.43–3.28)	< 0.001	95/21
	TnT, log	2.85 (1.76–4.61)	< 0.001	99/21
	GFR	0.96 (0.94–0.98)	< 0.001	102/21
	Age	1.06 (1.02–1.11)	< 0.001	105/21
	Sex, M vs F	7.72 (1.04–57.58)	0.046	105/21
	Group, ATTRv-PN vs ATTRwt	0.60 (0.18–1.97)	0.4	105/21
	Group, ATTRv-CM vs ATTRwt	0.61 (0.23–1.63)	0.33	
	MAPSE, log	0.46 (0.14–1.45)	0.18	93/21
	EDD	0.97 (0.90–1.05)	0.46	94/21
	HW	1.26 (1.14–1.41)	< 0.001	93/21
	Septum	1.16 (1.07–1.25)	< 0.001	94/21
	Left ventricular hypertrophy index	16.71 (4.2–66.3)	< 0.001	93/21
Death/HTX	Cell size (dichotom), high vs low	0.12 (0.02–0.58)	0.009	105/9
	NTproBNP, log	0.85 (0.57–1.27)	0.43	88/6
	NTproBNP, GFR adjust, log	1.66 (0.96–2.88)	0.07	95/9
	TnT, log	3.34 (1.31–8.52)	0.01	98/9
	GFR	0.93 (0.89–0.97)	0.002	102/9
	Age	1.04 (0.98–1.11)	0.21	105/9
	Sex, male vs female	2.41 (0.30–19.31)	0.1	105/9
	Group, ATTRv-PN vs ATTRwt	0.96 (0.17–5.48)	0.96	105/9
	Group, ATTRv-CM vs ATTRwt	0.75 (0.17–3.4)	0.71	
	MAPSE, log	2.30 (0.62–8.51)	0.21	93/9
	End diastolic diameter	0.99 (0.89–1.11)	0.93	94/9
	Posterior wall	1.10 (0.92–1.32)	0.31	93/9
	Intraventricular septum	1.13 (1.00–1.27)	0.054	94/9
	Left ventricular hypertrophy index	4.86 (0.38–62.5)	0.23	93/9

**Table 3:** Multivariate analyses for combined endpoint. *n* = 85/*n* events = 25

Feature	HR (95% CI)	*p* value
Cell size (dichotom), high vs low	0.31 (0.12–0.82)	0.02
NTproBNP, GFR adj, log	0.79 (0.98–3.27)	0.06
TnT, log	1.55 (0.8–2.99)	0.19
GFR	0.99 (0.96–1.01)	0.26
Age	0.96 (0.9–1.02)	0.17
Sex, male vs female	1.36 (0.25–7.5)	0.73
Posterior wall	1.04 (0.81–1.33)	0.77
Septum	0.98 (0.8–1.21)	0.84
LVHI	0.85 (0.01–80.37)	0.94
NYHA class		
II	0.74 (0.3–1.85)	0.52
III	0.89 (0.34–2.31)	0.81
IV	1.56 (0.2–12.35)	0.67

To mathematically maximize the observed trends under unstimulated condition (green arrow) and PE treated condition (red arrow), the hypertrophy index (HI) was calculated as change in cell size upon PE treatment normalized to the cell size in untreated condition. Control and ATTRv-PN plasma-treated cells showed strongest induction in hypertrophy without significant differences in HI between the two groups. HI with 5% plasma for control was 39 ± 2% (mean model estimate) and for ATTRv-PN HI was 36 ± 1% (*p* = 0.09). Interestingly, HI was significantly attenuated for ATTRv-CA (32 ± 1%; *p* < 0.001) and ATTRwt (28 ± 1%; *p* < 0.001) treated cells compared to control condition. In 20% plasma-treated cells, HI did not show any significant differences between controls (Ctrl) (16 ± 8%) and ATTRv-PN (12 ± 6%, *p* = 0.7), ATTRv-CA (18 ± 5%; *p* = 0.85) or ATTRwt (4 ± 5%; *p* = 0.21). Therefore, for further endpoint analysis in our study HI with 5% plasma conditions was chosen. Please note, there was no impact of medication upon HI as shown in supplementary Fig. 1 for diuretics, beta blockers, ACE/AT1 inhibitors, calcium channel blockers or tafamidis (Table 3).

### Attenuation of PE-induced cardiomyocyte hypertrophy in vitro allows prediction of future cardiovascular events

**Fig. 2:**
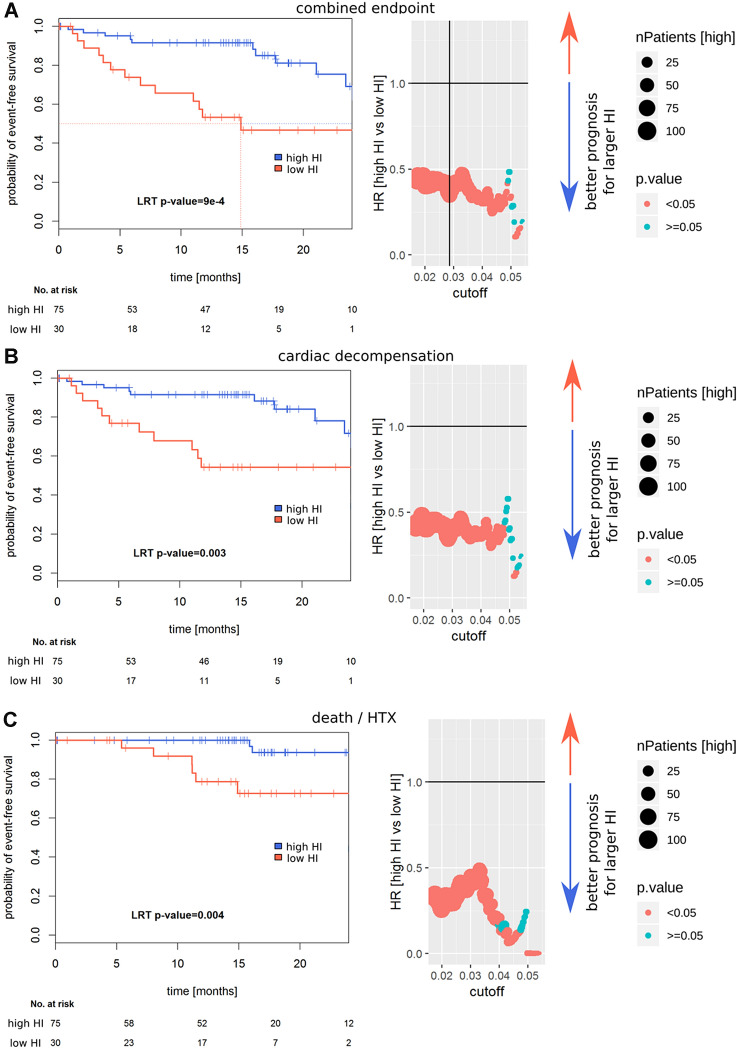
Prognostic value of HI. Left: Kaplan–Meier survival curves are shown; *p* values are calculated using a Cox-PH model. Right: Cutoff selection using Cox-PH models for the separation of prognostically different groups, the vertical line in **a** indicates the further utilized cutoff. Wald *p* values are reported. In **a** the combined endpoint (death, cardiac transplantation or decompensation), in **b** the endpoint decompensation and in **c** the endpoint death or HTX are depicted

PE-induced normalized cellular growth (HI) was analyzed using Cox proportional hazard models to identify cutoffs for the prognostic separation of patients (Fig. 2). The optimal HI was calculated as 2.9% (low HI group: *n* = 30, high HI group: *n* = 75). For all 89 patients follow-up after index event was available. Median follow-up was 14.8 [95% CI 14.2–16.9] months. Nine patients died, five were listed for high urgent heart transplantation and one patient was successfully transplanted. Twenty patients were hospitalized due to cardiac decompensation requiring additional treatment with diuretics for recompensation. Twenty-one patients reached the pre-specified combined endpoint (MACE). As shown in the Kaplan–Meier curves, patients with low hypertrophic response (low HI) had a higher risk for MACE (*p* < 0.001), decompensation (*p* = 0.003) and death/htx (*p* = 0.004).

### Attenuated PE-induced cellular growth allows risk prediction in cTnT and NTproBNP positive patients

**Fig. 3:**
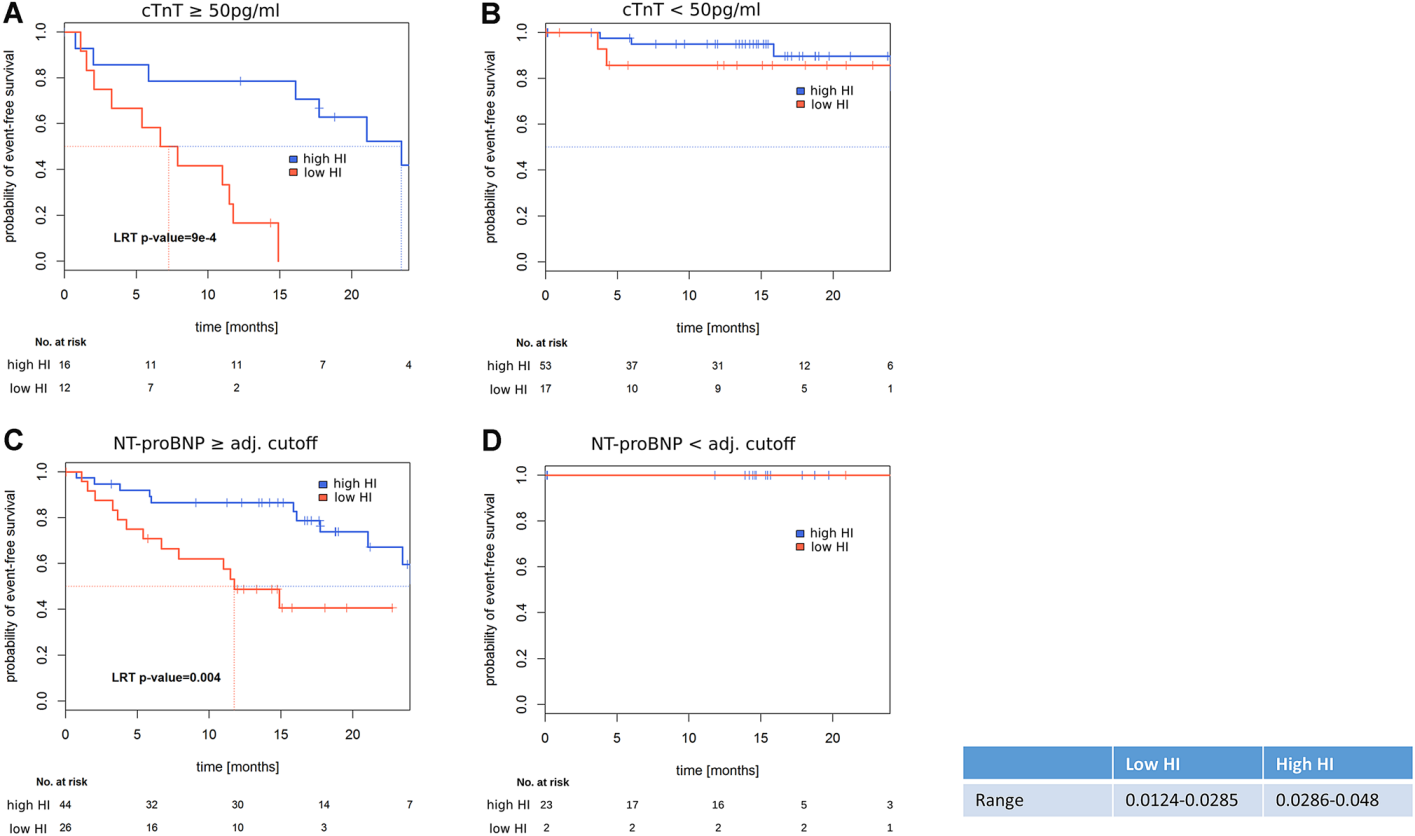
Time-to-event analyses in established biomarker (cTNT, NTproBNP) defined subcohorts. **a** TNT > 50 pg/ml, **b** TNT < 50 pg/ml, **c** NTpro-BNP, GFR adjusted ≥ patient specific cutoff, **d** NTpro-BNP, GFR adjusted < patient specific cutoff. Kaplan–Meier curves, log-rank *p* value of Cox-PH models as explained in Fig. 2

Two well-established biomarkers for risk stratification in ATTR are cardiac TroponinT (cTnT) and NTproBNP levels [7, 16]. Therefore, we repeated our risk assessment for patients in these two high-risk subgroups. Interestingly, no prediction for future MACE was possible: neither in the group of cTnT negative patients nor in the cohort of NTproBNP negative patients (Fig. 3b, d). In contrast, in the cTnT positive high-risk group (cTnT > 50 ng/ml) our novel in vitro hypertrophy index (HI) allowed further risk stratification (Fig. 3a; *p* = 9e−4). Also in the high-risk group of NTproBNP positive patients, a clear association with future MACE was observed (Fig. 3c; *p* = 0.004).

### Attenuated PE-induced hypertrophy is an independent risk factor

To estimate the value of the novel risk index for prediction of future events, we calculated the hazard ratios for established predictors as well as for the HI for MACE, decompensation and dead/htx using univariate Cox proportional hazard models. HI was significantly associated with all three pre-specified endpoints: MACE (*p* < 0.001), decompensation (*p* = 0.003) and dead/htx (*p* = 0.004). Additionally, prognosis was evaluated for NTproBNP, cTnT, GFR, age, gender, diagnosis (ATTRv-CA, ATTRv-PN, ATTRwt) and established echocardiographic parameters [end diastolic diameter (EDD), estimated heart weight (HW), septum thickness and left ventricular hypertrophy index (LVHI)].

To confirm independent risk prediction using HI, a multivariate cox regression analysis for MACE including parameters significant in the univariate analysis (both established biomarkers NTproBNP and cTnT, as well as GFR, age, gender, HW, septum thickness and LVHI) was performed. HI showed an independent association with risk with a hazard ratio of 0.31 [95% CI 0.12–0.82] (*p* = 0.02).

## Discussion

In the present work we established a novel functional biomarker to predict future cardiac events in patients with ATTR. Taking advantage of an automated high-throughput fluorescence microscope (InCell analyzer combined with the free available CellProfiler software), morphological analysis of NRCMs was performed in the presence of patient plasma or plasma from healthy controls. Cell size was quantified under basal condition or upon treatment with the hypertrophy-inducing agent phenylephrine—a well-established stimulus for basic research in vitro experiments [11, 12]. ATTR patients represent a heterogeneous group with primary cardiac, neuropathic or mixed clinical signs. Irrespective of the clinical presentation or underlying diagnosis (ATTRv or ATTRwt) attenuated hypertrophic response of cardiomyocytes upon PE treatment showed predictive value in terms of death, cardiac transplantation or decompensation. In contrast, no significant difference between the groups with or without PE stimulation was detectable at index event. Therefore, the diagnostic value of our assay is limited. In the study the significance of well-established predictors for outcome were confirmed. Cardiac TroponinT (cTnT), NTproBNP, kidney function, age, gender as well as echocardiographic parameters were associated with outcome as shown before [16, 28–33]. Strikingly, in the multivariate analysis the novel hypertrophy index (HI) was still significantly associated with the pre-specified combined endpoint (hazard ratios 0.31; *p* < 0.02). Interestingly, in our subgroup analysis HI had no additional prognostic value in the group of cTnT or NTproBNP negative patients confirming the significance of these biomarkers for risk stratification. In contrast, only in the cohort of patients with positive biomarkers HI had a strong additional value. Therefore, we speculate that the factors causing alteration in hypertrophic response rather precede or coincide with the myocardial stress and injury. And only if these so far unknown additional stress factors cause myocardial injury, the toxicity reaches a clinical relevant level mirrored in the prognosis. The underlying agent causing the toxicity is unknown and has to be the topic of future studies. In summary, HI allows further discrimination of the highest risk in the group of risk patients identified by established biomarkers cTnT and NTproBNP.

The work represents a pilot study with a monocentric prospective design. Confirmation of the herein results in an independent cohort is needed to open the assay for broader application. Primary rat cardiomyocyte preparation is a sensitive procedure with significant inter-laboratory variability. However, normalization of the PE induced response to the basal condition expressed as hypertrophy index (HI) will allow comparability between different laboratories. In contrast to other so-far established biomarkers quantified in absolute numbers for their concentration, the HI represents a functional novel innovative approach integrating the significance of multiple agents in the plasma of patients into one read-out parameter. The exact underlying mechanism for altered hypertrophic response in our assay remains cryptic, while it can be speculated that the amyloid itself, catecholamines, inflammatory markers, antibodies, effects by cellular immunity or other not yet identified mediators will affect the final read-out [4, 5, 34–37].

The heart is a post-mitotic organ and the primary response to increased workload or stress is cellular cardiomyocyte hypertrophy. This initially physiologic adaption is known to result in a vicious circle leading to pathological hypertrophy when sustained chronically. Over time diastolic function is impaired and symptoms of heart failure develop [6]. In echocardiography, increased wall thickness and diastolic dysfunction become evident [30]. Intense histological analysis of cardiac biopsies revealed amyloid load as prognostic determinant in cardiac amyloidosis; the extent of hypertrophy on the cellular level has not been analyzed in detail yet [38]. Although treatment of cardiomyocytes in vitro with patient plasma induced increase in cell size, no significant difference was measurable between groups. Only a trend was seen from healthy controls over ATTRv-PN and ATTRv-CA to ATTRwt showing minimal increase in cell size. Also after PE treatment cell sizes of the groups were not significantly different, a trend was notable for impaired hypertrophic growth in ATTRwt patients and best response in healthy controls or ATTRv-PN. Based on this observation the hypertrophy index was calculated to mathematically maximize this effect of both trends.

Recent pharmaceutical developments are promising for causal treatment in ATTR pathology [39–41]; however, the decision, which of these bio-pharmaceuticals (siRNA or tetramer stabilizers) are ultimately prescribed depends on the individual decision of the treating physician. Therefore, for better risk stratification and resource allocation meaningful prognostic markers are needed. Furthermore, it might be possible to monitor with the herein presented functional assay the drug efficacy and tailor individually the proper dosage of these expensive substances in the future. Also for cardiac transplantation additional risk predictors will be helpful since number of available organs is limited. Better estimation of prognosis for this cohort is needed since the classical parameters for organ allocation are not necessarily altered: systolic function might be preserved and cardiac index, e.g. only slightly reduced and yet prognosis is poor [42, 43].

## Limitations

In the present study, we used an in vitro hypertrophy assay that requires laboratory facilities, technical instruments and the possibility to process data sets requiring high computing capacity that excel personal computer performs. In our study normalized changes in cell size depicted as HI allowed excellent additional risk stratification in biomarker positive patients. Absolute cell sizes may vary between laboratory facilities and very clearly will depend on NRCM preparation. Therefore, the proposed assay might only be useful for specialized centers possessing an appropriate technical infrastructure, e.g. tertial referral amyloidosis centers. Since plasma samples can be stored and shipped under appropriate cooled conditions, this approach might still find broad application.

In patients with negative cardiac biomarkers, our in vitro assay did not allow additional prognostic assessment. This might be due to the small sample size of this cohort and of course the low incidence of clinical events in the low-risk biomarker negative group. Yet our assay could measure differences in this cohort and the clinical relevance thereof has to be clarified in future studies with longer follow-up time and higher numbers of patients with low risk according established stratification strategies.

## Conclusions

Non-invasive prognostic markers in ATTR are limited. Our in vitro hypertrophy assay represents an innovative novel approach for risk prediction taking advantage of an in vitro biological response of cardiomyocytes towards patient plasma. The HI adds strong independent information additionally to established biomarkers, namely cTNT and NTproBNP as well as clinical data (age, gender, kidney function and echocardiographic data) for patients at highest risk with ATTR. These results need to be confirmed in larger cohorts.

## Supplementary Information

Below is the link to the electronic supplementary material.

Supplementary file1 (TIF 700 KB)

Supplementary file2 (TIF 1581 KB)

## Data Availability

Software applications are declared within the text. Custom codes are available on demand.
