# Supplementary figures and images for: Impaired in vitro growth response of plasma-treated cardiomyocytes predicts poor outcome in patients with transthyretin amyloidosis
Source: Clin Res Cardiol. 2021 Jan 22;110(4):579–90. doi: 10.1007/s00392-020-01801-y (PMC8055573; doi:10.1007/s00392-020-01801-y)

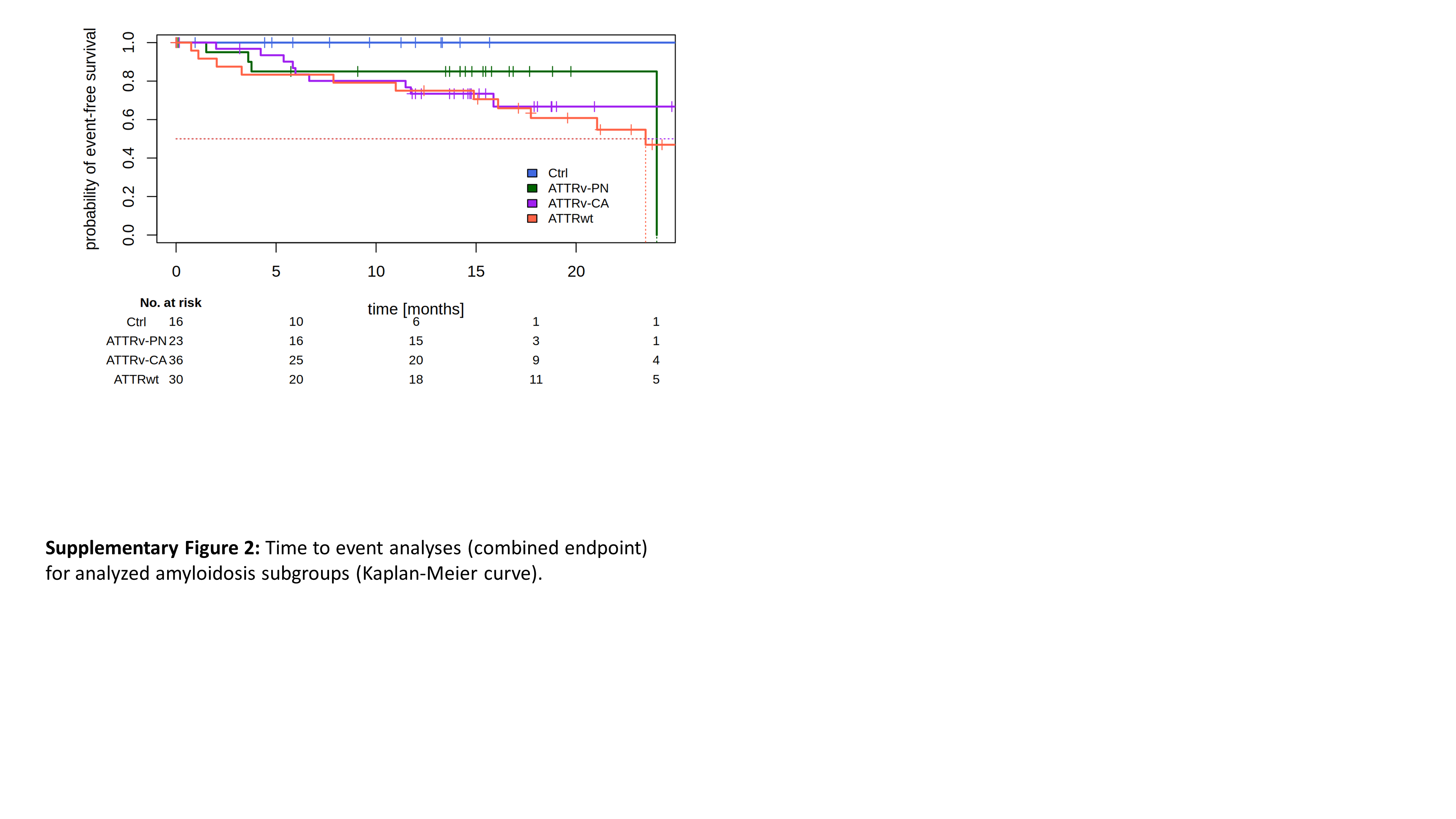

Supplement: Supplementary file 1 — Supplementary file1 (TIF 700 KB) [file 392_2020_1801_MOESM1_ESM.tif]

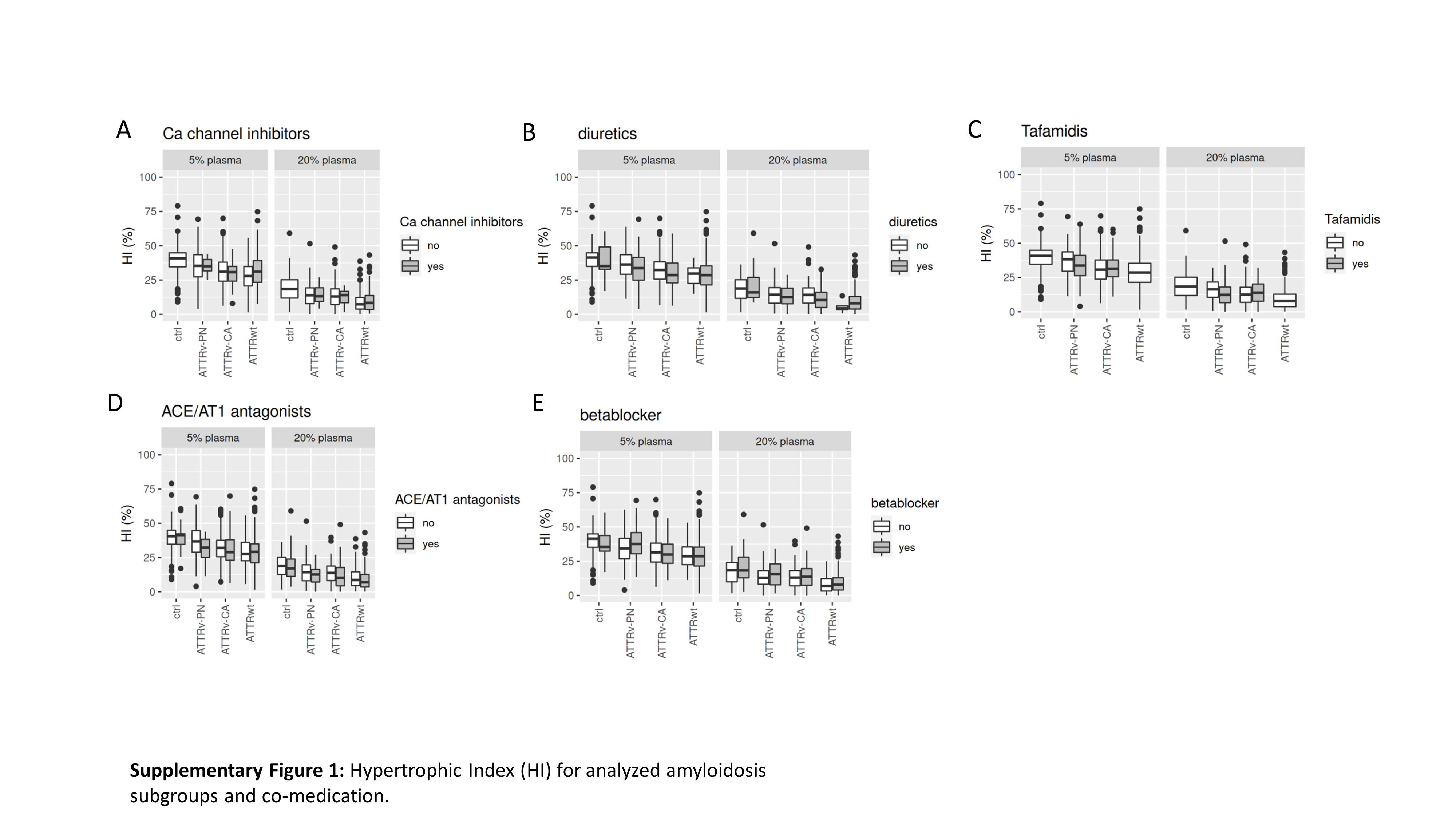

Supplement: Supplementary file 2 — Supplementary file2 (TIF 1581 KB) [file 392_2020_1801_MOESM2_ESM.tif]
